# The Therapeutics of Nature

**Published:** 1845-03

**Authors:** 


*The Therapeutics of Nature.*—"Theraputics, it is very generally admitted, is one of those branches of medical knowledge in which there exists the greatest amount of errors, defects, and prejudices; and where experience is alike most difficult and deceptive. The mistakes, that are daily made, are often far greater than we are willing to admit. And then, how little do we know of the extent of Nature's own curative resources, and how much she will often effect, unaided by, or perhaps even in spite of, the interference of art. In the practice of our profession, it should ever be borne in mind that we have to do, not only with the existing disease, but also with the conservative and reparatory efforts of Nature,—which, by itself, is often sufficient to produce a cure. Hence, those reputations of medicines and modes of treatment, which so rapidly start up and are as quickly forgotten; and hence those false-gods of Therapeutics that to-day are adored, and to-morrow are despised."—*Med. Chir. Rev. from Bull. de Therap.*

